# Quality of life outcomes in two phase 3 trials of setmelanotide in patients with obesity due to LEPR or POMC deficiency

**DOI:** 10.1186/s13023-022-02186-z

**Published:** 2022-02-05

**Authors:** Peter Kühnen, Martin Wabitsch, Julia von Schnurbein, Costel Chirila, Usha G. Mallya, Patrick Callahan, Ari Gnanasakthy, Christine Poitou, Philipp M. Krabusch, Murray Stewart, Karine Clément

**Affiliations:** 1 Charité - Universitätsmedizin Berlin, Corporate Member of Freie Universität Berlin und Humboldt-Universität zu Berlin, Institute for Experimental Pediatric Endocrinology, CVK, Augustenburger Platz 1, 13353 Berlin, Germany; 2 Division of Pediatric Endocrinology and Diabetes, Center for Rare Endocrine Diseases, Department of Pediatrics and Adolescent Medicine, University of Ulm, Eythstraße 24, 89075 Ulm, Germany; 3 RTI Health Solutions, 3040 East Cornwallis Road, P.O. Box 12194, Research Triangle Park, NC 27709-2194 USA; 4 Rhythm Pharmaceuticals, Inc., 222 Berkeley Street, Suite 1200, Boston, MA 02116 USA; 5 Genesis Research, 111 River Street, Suite 1120, Hoboken, NJ 07030 USA; 6 Nutrition Department, Assistance Publique Hôpitaux de Paris, Pitié-Salpêtrière Hospital, IE3M Building, Room 637, 6th floor, 91 boulevard de l’Hôpital, 75013 Paris, France; 7 INSERM, Nutrition and Obesity; Systemic Approaches (NutriOmique) Research Group, Sorbonne University, Room 637, 6th floor, 91 boulevard de l’Hôpital, 75013 Paris, France

**Keywords:** Hyperphagia, IWQOL-Lite, PedsQL, PHQ-9, Rare genetic diseases of obesity, Disease burden, Melanocortin receptor

## Abstract

**Introduction:**

Individuals with proopiomelanocortin (POMC) or leptin receptor (LEPR) deficiency are young and experience severe obesity, hyperphagia, and comorbidities, which can impair quality of life (QOL).

**Methods:**

Two pivotal Phase 3 trials explored the effect of setmelanotide on body weight and hunger in individuals with obesity due to POMC (NCT02896192) or LEPR (NCT03287960) deficiency. QOL and depression were investigated in parallel using the disease-specific, age-appropriate Impact of Weight on Quality of Life-Lite (IWQOL-Lite), Pediatric Quality of Life Inventory (PedsQL), and Patient Health Questionnaire-9 (PHQ-9).

**Results:**

In total, the POMC and LEPR trials enrolled 21 patients. Adults (≥ 18 years old; n = 7) had moderate-to-severe impairment in QOL at baseline, with mean (standard deviation [SD]) IWQOL-Lite total score 60.3 (13.2; maximum IWQOL-Lite total score = 100). The effect of setmelanotide on IWQOL-Lite total score was observed as soon as Week 5. Among those with scores at Week 52, 5 of 6 adults experienced a clinically meaningful improvement, with mean (SD) total scores increased from baseline by 24.2 (12.1) points. Children (6–12 years old; n = 2) and adolescents (13–17 years old; n = 4) had impaired QOL at baseline, with mean (SD) self-reported PedsQL total scores 53.3 (6.2) and 63.3 (29.1), respectively (maximum PedsQL total score = 100). Three of 5 patients experienced clinically meaningful improvement in PedsQL, with 2 children whose PedsQL total score increased by 28.3 and 3.3 points and 3 adolescents whose mean (SD) total score increased from baseline by 5.8 (18.3) points. Baseline mean (SD) PHQ-9 score (in those ≥ 12 years old) was 5.3 (3.8) and was generally maintained through Week 52.

**Conclusions:**

Patients with POMC or LEPR deficiency had impaired, and in some cases severely impaired, QOL before setmelanotide treatment. Setmelanotide improved QOL in patients as early as Week 5, with some patients no longer experiencing impaired QOL at Week 52. Improvements in QOL may be related to a reduction in hunger and body weight associated with setmelanotide. Because of the highly complex psychological consequences of rare genetic diseases of obesity, some patients may require a long period of treatment to improve QOL and benefit from interdisciplinary care.

## Background

Obesity can negatively impact an individual’s life through increased risk of morbidity (e.g., cancer, respiratory disease, type 2 diabetes mellitus, and cardiovascular disease), the burden of social stigma, and impaired quality of life (QOL) [1–6]. These burdens do not exist in isolation, but rather, impaired health status and social stigma can impact an individual’s QOL [1, 7]. Individuals with obesity are approximately twice as likely to have health concerns, chronic diseases, physical disability, or limitations to their daily life activities compared with nonobese individuals [7]. Children or adolescents with obesity were 5.5 times more likely to have an impaired QOL than a control population [6]. QOL in patients with obesity declines with higher grades of obesity [8]. The risk for impaired QOL and mental health is most pronounced with severe obesity [7, 9–11]. Individuals with severe obesity were 4 times as likely to have limitations to their daily life activities and were 30% more likely (odds ratio, 1.3) to have depression compared with nonobese individuals. [7, 10].

In addition to socioeconomic, environmental, and cultural factors, obesity can also be driven by genetics [12, 13]. The leptin-melanocortin signaling pathway regulates hunger and energy balance, and disrupted activity of this pathway through genetic variants may cause insatiable hunger, known as hyperphagia [14, 15]. Such genetic variants in the leptin-melanocortin signaling pathway are the cause of rare genetic diseases of obesity, including proopiomelanocortin (POMC) deficiency and leptin receptor (LEPR) deficiency, which are characterized by early-onset severe obesity and hyperphagia [13, 14, 16]. Hyperphagia can be a daily struggle for patients, placing burdens on them, their families, and their caregivers [17, 18]. A survey of individuals with rare genetic diseases of obesity found that 45% reported fear or anxiety over creating stress for their support system. Individuals with POMC and LEPR deficiency experience comorbidities, including diabetes mellitus, frequent infections, and hormonal insufficiencies, which alone can impair QOL [14, 16, 19, 20]. Together, hyperphagia, the accompanying obesity, and associated comorbidities can contribute to physiologic and psychological impairments and reduced QOL in patients with POMC or LEPR deficiency [18]. Given the early-onset of obesity in these populations, patients are often young and may have been living with these burdens most of their lives [20–22].

Very little research exists on the impact of POMC or LEPR deficiency on QOL. There is an unmet need to evaluate whether weight loss therapeutics reduce the burdens of hunger and obesity and improve QOL, especially in genetic obesity. In Phase 3 clinical trials, the melanocortin-4 receptor agonist setmelanotide significantly reduced weight and hunger in patients with obesity due to POMC or LEPR deficiency, as reported by Clément et al. [20] Patients in the POMC and LEPR trials experienced mean (standard deviation [SD]) weight loss of 25.6% (9.9%; *P* < 0.0001) and 12.5% (8.9%; *P* < 0.0001), respectively, and mean (SD) hunger reduction of 27.1% (28.1%; *P* = 0.0005) and 43.7% (23.7%; *P* < 0.0001), respectively, after ~ 52 weeks of setmelanotide [23]. For this reason, the aim of this substudy was to evaluate if the reduction in body weight and hunger scores was accompanied by improvement in the QOL of these patients with monogenic obesity.

## Methods

### Study design

Two pivotal Phase 3, single-arm, open-label trials with a double-blind placebo-controlled, withdrawal period investigated the effect of setmelanotide on body weight in individuals with obesity due to POMC or LEPR deficiency. Patients for each study were identified by the investigators from clinical site databases or genetic obesity registries [20]. The POMC trial (NCT02896192) had active sites in Germany, France, Canada, the United States, Spain, and Belgium. The LEPR trial (NCT03287960) had active sites in Germany, France, the United Kingdom, and The Netherlands.

The POMC trial enrolled patients ≥ 6 years old with homozygous or compound heterozygous *POMC* or *PCSK1* variants. The LEPR trial enrolled patients ≥ 6 years old with homozygous or compound heterozygous *LEPR* variants. For inclusion in either trial, obesity was defined as body mass index (BMI) ≥ 30 kg/m^2^ for adults ≥ 18 years old and BMI ≥ 95th percentile for age on growth chart assessment for children or adolescents 6–17 years old. Patients were excluded from either trial if they reported moderately severe or severe depression (Patient Health Questionnaire-9 [PHQ-9] score ≥ 15) or suicidal ideation (type 4 or 5 on the Columbia Suicide Severity Rating Scale), recent suicidal behavior, or history of suicide attempt.

**Fig. 1:**
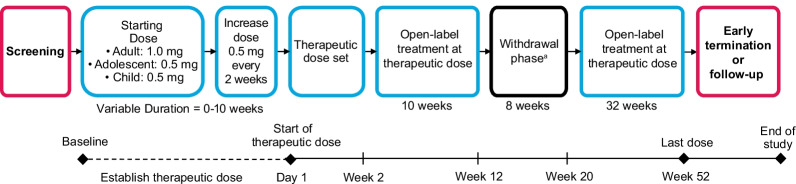
Study design. ^a^Withdrawal phase included a variably timed 4-week placebo period and a 4-week period of setmelanotide treatment

The trials began with an open-label dose titration phase, during which adult and child/adolescent patients initially received doses of 1.0 and 0.5 mg, respectively (Fig. 1). Active dose is considered any dose of setmelanotide received. Doses were up-titrated 0.5 mg every 2 weeks until the therapeutic dose (defined as the dose of setmelanotide associated with 2–3-kg weight loss per week in adults and 1–2-kg weight loss per week in children/adolescents, and hunger reduction) was established. The last 2 weeks of the dose titration phase were considered the first 2 weeks of open-label treatment. Patients continued open-label active treatment for an additional 10 weeks. Patients who experienced ≥ 5-kg weight loss (or ≥ 5% weight loss for patients with baseline body weight < 100 kg) continued into the 8-week double-blind, placebo-controlled withdrawal phase, which included 4 weeks of placebo. Patients then completed an additional 32 weeks of open-label active treatment for a total of ~ 1 year of therapeutic dosing.

Because of the rarity of these diseases, a small sample size (~ 10 patients) was planned for each trial. The pivotal cohort of patients used for the analysis was the first ~ 10 patients enrolled in each trial. Enrollment remained open for additional supplementary patients, but the results from this supplementary cohort are not reported here. The full analysis set (FAS) comprised patients who received ≥ 1 dose of setmelanotide and completed the baseline assessment. The designated use set (DUS) comprised patients who received ≥ 1 dose of setmelanotide, experienced ≥ 5-kg weight loss (or ≥ 5% weight loss for those with baseline body weight < 100 kg), and continued into the double-blind, placebo-controlled withdrawal phase. Data for the QOL and depression endpoints were analyzed for the DUS population at Week 52 to align with the efficacy analysis in Clément et al. [20] Because of the small population sizes and clinical relevance of POMC and LEPR deficiency, results from the patient populations combined across both trials are reported.

Both trials were conducted in accordance with the International Council on Harmonisation for Good Clinical Practice and the Declaration of Helsinki. Institutional review board or independent ethics committee approval was obtained at all trial sites. Patients provided informed consent. Patient confidentiality was maintained.

### Assessments

Body weight, height, BMI, and hunger were assessed throughout the trials. Age-appropriate questionnaires were used in the assessment of hunger, QOL, and depression when available. The primary endpoint, which was the proportion of patients who reached ≥ 10% loss in body weight after ~ 52 weeks (with variance due to scheduling), was met by 80% of patients in the POMC trial and 45% of patients in the LEPR trial, as reported in Clément et al. [20] Hunger was assessed using a daily questionnaire, which participants responded to in the morning before eating or receiving setmelanotide. Patients ≥ 12 years old responded to a set of 3 questions using a Likert-type numerical rating scale ranging from 0 to 10, where 0 = not hungry at all and 10 = hungriest possible. The 3 questions were, “In the last 24 hours, on average, how hungry did you feel?”, “In the last 24 hours, how hungry did you feel when you were the most hungry?”, and “This morning when you woke up for the day, how hungry did you feel?” Patients 6–11 years old responded to a single question, “How hungry do you feel right now?”, using a 5-point scale consisting of faces and verbal descriptors. The exploratory analyses performed in this study were mostly descriptive in nature.

QOL in patients was assessed using the Impact of Weight on Quality of Life-Lite (IWQOL-Lite) in patients ≥ 18 years old. The IWQOL-Lite is a 31-item obesity-specific measure of health-related QOL that consists of a total score and scores on each of 5 domains (physical function, self-esteem, sexual life, public distress, and work) [24]. Similarly, QOL in patients < 18 years old was assessed using the age-appropriate Pediatric Quality of Life Inventory (PedsQL). Children responded to the young child report (6–7 years old) or child report (8–12 years old). Adolescents (13–17 years old) responded to the teen report. The PedsQL is an age-dependent, self-reported (or parent-reported) measurement system for assessing health-related QOL in children and adolescents who are healthy or experiencing acute or chronic health conditions [25]. QOL questionnaires were administered in each trial according to the following schedule: at screening and before treatment administration at visits 4 (week 5), 6 (week 13), 9 (week 27), 11 (week 39), and 13 (week 53). In instances when the incorrect patient-reported outcome instrument, by age, was administered to a patient, the data were not included in the analysis. Hunger questionnaires, IWQOL-Lite or PedsQL, and the PHQ-9 were administered at appropriate visits before treatment administration in each trial.

Raw scores from both the IWQOL-Lite and PedsQL are transformed onto a scale of 0 to 100, such that 0 represents the worst possible QOL and 100 represents the best possible QOL [26, 27]. The mean (SD) IWQOL-Lite total score of a comparative population of normal and overweight individuals is 94.7 (7.6) [26]. Severity of impairment for IWQOL-Lite is based on the number of SDs below the comparative mean, such that total scores of 87.1–94.6 (< 1 SD), 79.5–87.0 (1 to < 2 SD), 71.9–79.4 (2 to < 3 SD), and < 71.8 (≥ 3 SD) represent no, mild, moderate, and severe impairment. Threshold values for clinically meaningful change in IWQOL-Lite scores are dependent on baseline scores and were referenced against those previously reported by Crosby et al. [26] The mean (SD) self-reported PedsQL total score for children and adolescents of a comparative population without obesity was 83.0 (14.8) [6]. In this study, impairment for PedsQL was defined as 1 SD below the comparative mean. For patient-reported scores, the clinically meaningful change was defined as 4.4, 6.7, and 5.3 for total score, physical health, and psychosocial health, respectively [27].

Depression was also assessed throughout the trials using the PHQ-9. The PHQ-9 is a self-reported 9-item depression scale for assessment of patients ≥ 12 years old. Depression severity is defined as minimal, mild, moderate, moderately severe, or severe for scores of 0–4, 5–9, 10–14, 15–19, and 20–27, respectively [28]. For PHQ-9, clinically meaningful change was defined as change in score of ≥ 5 points [29]. If a patient had a PHQ-9 score ≥ 10 during the trial, they were referred to a mental health professional.

## Results

### Participant demographics

A total of 21 patients were enrolled in the pivotal cohorts of the POMC and LEPR trials, with mean (SD) age and BMI of 21.2 (7.7) years and 44.5 (10.4) kg/m^2^, respectively; 16 patients were included in the DUS population (patients who experienced ≥ 5-kg weight loss [or ≥ 5% weight loss for those with baseline body weight < 100 kg]). In the DUS population, patients were aged 11–37 years old; 2 patients were 6–12 years old, 7 patients were 13–17 years old, and 7 patients were ≥ 18 years old.

### Baseline IWQOL-Lite scores and changes with setmelanotide (in adults)

At baseline, 7 adult patients reported a IWQOL-Lite total score ranging from 43 to 77; 5 had severe impairment [26] (defined as total score < 71.8; Table 1) and 2 had moderate impairment [26] (defined as total score in the range of 71.9–79.4). The mean (SD) baseline IWQOL-Lite total score was 60.3 (13.2).

**Table 1:** IWQOL-Lite Summary (Age ≥ 18 Years)

	Patient 1^a^	Patient 2^a^	Patient 3^a^	Patient 4^b^	Patient 5^b^	Patient 6^b^	Patient 7^b^	Mean (SD)
IWQOL-Lite total score at baseline	50	43	70	74	77	52	56	60.3 (13.3)
Change in IWQOL-Lite total score at Week 52	44	31	17	23	21	–	9	24.2 (12.1)
Relevant improvement cutoffs for IWQOL-Lite total score [26]^c^	12.0	12.0	12.0	8.3	8.2	12.0	12.0	–
Percent change in IWQOL-Lite total score at Week 52	88.0	72.1	24.3	31.1	27.3	–	16.1	43.1 (29.4)
Change in domain scores at Week 52								
PF	34	25	4	18	28	–	0	18.2 (13.6)
SL	50	–	69	0	–	–	–	39.7 (35.6)
W	63	38	19	62	19	–	7	34.7 (23.7)
PD	40	30	0	10	25	–	20	20.83 (14.3)
SE	47	39	18	29	7	–	14	25.7 (15.4)
Body weight, percent change	− 34.8	− 25.8	− 27.7	− 21.0	− 15.6	–	− 2.3	− 21.2 (11.3)
Most hunger, percent change	− 14.3	− 5.8	− 72.2	− 64.3	− 66.7	–	− 37.5	− 43.5 (28.7)

IWQOL-Lite, Impact of Weight on Quality of Life-Lite; LEPR, leptin receptor; PD, public distress; PF, physical functioning; POMC, proopiomelanocortin; SD, standard deviation; SE, self-esteem; SL, sexual life; W, work

^a^Patient enrolled in POMC trial

^b^Patient enrolled in LEPR trial

^c^Improvement and deterioration cutoff scores are dependent on baseline IWQOL-Lite total scores

**Fig. 2:**
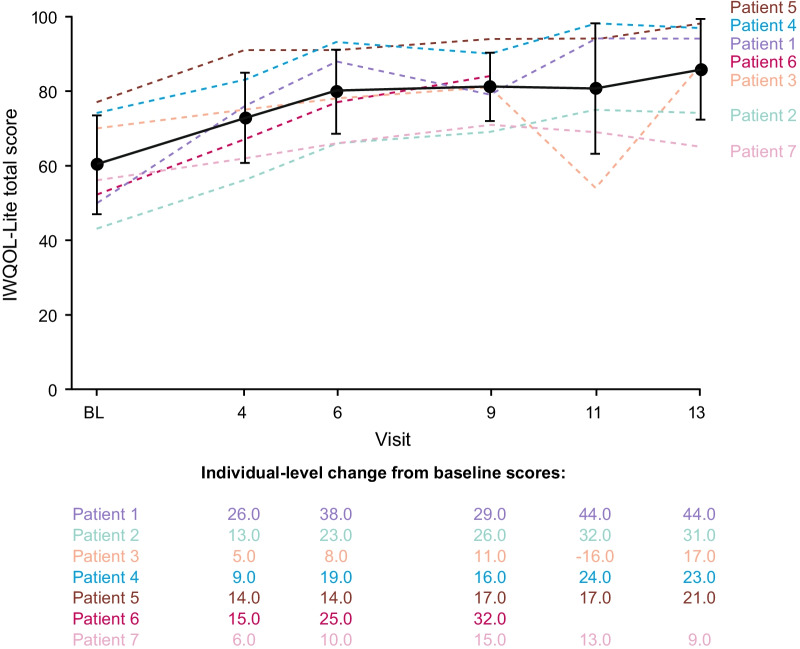
Impact of Weight on Quality of Life-Lite (IWQOL-Lite) total score in patients ≥ 18 years old. Broken lines represent individual patient data. Solid line represents the mean. Error bars represent the standard deviation. *BL* baseline

Of the 7 adults in the DUS pivotal cohort, 6 reported IWQOL-Lite scores at Week 52. Patient scores improved 9–44 points at Week 52, with mean (SD) change from baseline at Week 52 of 24.2 (12.1). Clinically meaningful improvement was experienced by 5 of 6 (83.3%) patients. Improvement cutoffs (ranging from change in scores of 8–12) are dependent on individual baseline IWQOL-Lite total scores [26]; relevant cutoffs are shown in Table 1. Improvement in IWQOL-Lite total score was seen as early as 5 weeks and was sustained through ~ 52 weeks (Fig. 2). The 2 adults with the lowest baseline QOL scores experienced the greatest improvement in QOL, improving from severely impaired to no or moderate impairment. One of these adults also experienced the greatest percent change in body weight among those reporting IWQOL-Lite scores. Improvements in each domain score were observed for most patients at Week 52.

### Baseline PedsQL scores and changes with setmelanotide (in children and adolescents)

At baseline, 2 children reported PedsQL total scores of 48.9 and 57.6; both had clinically meaningful impairment [6] (defined as total score < 68.2; Table 2). Also at baseline, 4 adolescents reported a PedsQL total score ranging from 23.9 to 85.9; 2 had clinically meaningful impairment (Table 3). The mean (SD) baseline PedsQL total score was 53.3 (6.2) and 63.3 (29.1) for children and adolescents, respectively.

**Table 2:** PedsQL summary (age 8–12 years; child reported)

	Patient 8^a^	Patient 9^a^	Mean (SD)
PedsQL total score at baseline	48.9	57.6	53.3 (6.2)
Change in PedsQL total score at Week 52	28.3	3.3	15.8 (17.7)
Percent change in total PedsQL score at Week 52	57.8	5.7	31.7 (36.8) *P* = 0.0311
PF	34.4	− 9.4	12.5 (30.9)
PH	25.0	10.0	17.5 (10.6)
Body weight, percent change	− 20.1	− 2.4	− 11.2 (12.5)

PedsQL, Pediatric Quality of Life Inventory; PH, psychosocial health; PF, physical functioning; POMC, proopiomelanocortin; SD, standard deviation

^a^Patient enrolled in POMC trial

**Table 3:** PedsQL summary (age 13–17 years; adolescent reported)

	Patient 10^a^	Patient 11^a^	Patient 12^a^	Patient 13^a^	Mean (SD)
PedsQL total score at baseline	23.9	85.9	84.8	58.7	63.3 (29.1)
Change in PedsQL total score at Week 52	21.7	− 14.1	9.8	–	5.8 (18.3)
Percent change in PedsQL total score at Week 52	90.9	− 16.5	11.5		28.7 (55.7) *P* = 0.0106
PF	21.9	− 18.8	15.6	–	6.3 (21.9)
PH	21.7	− 11.7	6.7	–	5.6 (16.7)
Body weight, percent change	− 26.2	− 30.2	− 35.6	− 27.3	− 29.8 (4.2)
Most hunger, percent change	− 54.7	− 37.5	− 1.4	− 3.5	− 24.3 (26.2)

PedsQL, Pediatric Quality of Life Inventory; PH, psychosocial health; PF, physical functioning; POMC, proopiomelanocortin; SD, standard deviation

^a^Patient enrolled in POMC trial

**Fig. 3:**
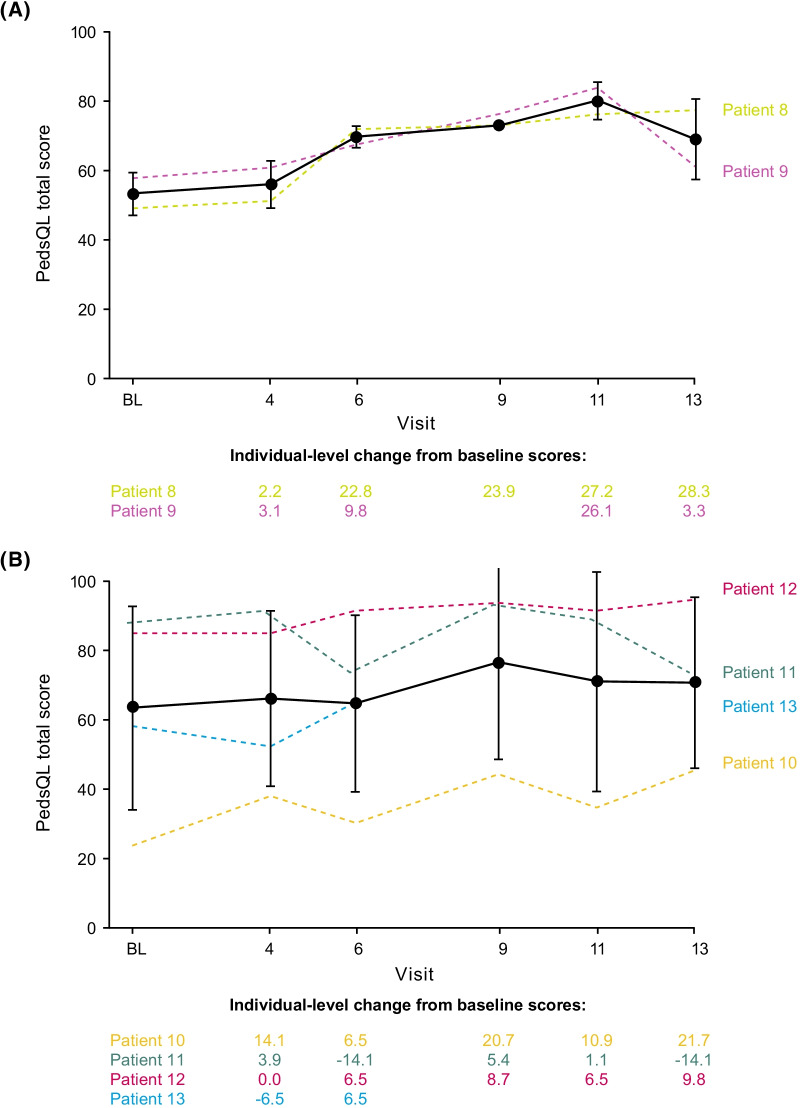
Pediatric Quality of Life Inventory total score in patients aged **a** 8–12 and **b** 13–17 years. Broken lines represent individual patient data. Solid lines represent the mean. Error bars represent the standard deviation. *BL* baseline

Both children in the DUS pivotal population reported PedsQL scores at Week 52. Patient total scores improved by 28.3 and 3.3 points at Week 52. Clinically meaningful improvement [27] (defined as total score change > 4.4) was experienced by 1 patient (Fig. 3). Of the 7 adolescents in the DUS pivotal cohort, 3 completed both the baseline and Week-52 PedsQL assessment. Patient total scores changed by a range of − 14.1 to 21.7 points at Week 52, with mean (SD) change from baseline at Week 52 of 5.8 (18.3). Clinically meaningful improvement was experienced by 2 of 3 patients (67%), and clinically meaningful worsening was experienced by 1 patient. Of the 5 children and adolescents who had scores reported at Week 52, 3 reported improvements in physical functioning scores (defined as score change > 6.7) and 4 reported improvements in psychosocial health scores (defined as score change > 5.3), secondary analyses of PedsQL [27]. Because children and adolescents are still growing, it is difficult to assess improvement in QOL relative to weight loss. However, the child and adolescent with the lowest baseline PedsQL total scores experienced the greatest improvements with setmelanotide.

### Baseline PHQ-9 scores and changes with setmelanotide

Baseline PHQ-9 scores ranged from 0 to 11, with 8, 3, and 3 patients reporting minimal, mild, and moderate depression, respectively (Table 4) [28]. The mean (SD) baseline PHQ-9 score was 5.3 (3.8). In the POMC and LEPR trials, PHQ-9 scores were generally maintained from baseline through Week 52, with mean (SD) change from baseline at Week 52 of − 0.5 (3.0). Two patients experienced clinically meaningful improvement [29] (defined as change ≥ 5 points) in PHQ-9. No patients experienced worsening depression.

**Table 4:** PHQ-9 summary (age ≥ 12 years)

	Patient 1^a^	Patient 2^a^	Patient 3^a^	Patient 4^b^	Patient 5^b^	Patient 6^b^	Patient 7^b^	Patient 10^a^	Patient 11^a^	Patient 12^a^	Patient 13^a^	Patient 14^b^	Patient 15^b^	Patient 16^b^	Mean (SD)
PHQ-9 score at baseline	11	10	8	9	4	5	2	11	4	3	3	3	1	0	5.3 (3.8)
Change in PHQ-9 score at Week 52	− 6	4	1	2^c^	0^d^	–	0	− 7	− 1	− 2	2	0	0^e^	0	− 0.5 (3.0)
Percent change in PHQ-9 score at Week 52	− 54.6	40.0	12.5	22.2	0.0	–	0.0	− 63.6	− 25.0	− 66.7	66.7	0.0	0.0	0.0	− 5.3 (39.2)

PHQ-9, Patient Health Questionnaire-9; SD, standard deviation

^a^Patient enrolled in POMC trial

^b^Patient enrolled in LEPR trial

^c^Unscheduled visit at day 393

^d^Unscheduled visit at day 351

^e^Unscheduled visit at day 393

## Discussion

POMC deficiency and LEPR deficiency are caused by genetic variants in the leptin-melanocortin signaling pathway and are characterized by early-onset severe obesity and hyperphagia [13, 14, 16]. Obesity can burden individual patients and caregivers, and obesity with genetically driven hyperphagia may further compound that burden [1, 2, 9, 17]. Obesity increases an individual’s risk of morbidity, social stigma, and impaired QOL [1–6, 9]. Hyperphagia, or insatiable hunger, and comorbidities can also lead to an impaired QOL [17–19]. Genetic testing is important in individuals with early-onset severe obesity and hyperphagia to identify those with rare genetic diseases of obesity [22]. This can lead to targeted therapies and allow patients to avoid additional burdens of frustration and medical complications that can be caused by failed weight loss interventions [22]. Weight loss and hunger reduction are important for individuals with obesity and hyperphagia because they can lead to improvements in QOL [26, 30].

The baseline characteristics of patients who enrolled in these two Phase 3 trials suggest that individuals living with rare genetic diseases of obesity, such as POMC or LEPR deficiency, have an impaired, and in some cases severely impaired, QOL. Setmelanotide was previously shown to reduce body weight and hunger scores in patients with these rare genetic diseases of obesity [20]. Clément et al. reported 80% and 45% of patients met the primary endpoint in the POMC and LEPR trials, respectively. Mean (SD) weight loss was − 25.6% (9.9%) in the POMC trial and − 12.5% (8.9%) in the LEPR trial (DUS; mean (SD) weight loss of 23.1% (12.1%) in the POMC trial and 9.7% (8.8%) in the LEPR trial for the FAS) [20, 23]. In the current analysis, setmelanotide was generally associated with a tendency toward improvements in disease-specific QOL measures, which may be related to decreases in hunger and, subsequently, body weight, although the small population size precluded meaningful correlation analyses.

Baseline IWQOL-Lite total scores suggest that some adults with severe obesity due to POMC or LEPR deficiency experience a lower QOL than individuals with general obesity [31]. Setmelanotide was associated with improvements in IWQOL-Lite total scores and domain scores in patients with these rare genetic diseases of obesity. Clinically meaningful changes were observed for 83% of adults with scores at Week 52; for 4 of 6 adults, these improvements led to no or mild impairment in QOL after setmelanotide treatment. Compared with a separate population of adults with severe obesity, setmelanotide treatment may result in an equal or greater proportion of patients who achieve clinically meaningful improvement in IWQOL-Lite total scores compared with lifestyle intervention or surgery for weight loss. Warkentin et al. demonstrated a clinically meaningful improvement in IWQOL-Lite total scores in 49% of 200 patients who underwent medically managed lifestyle counseling for weight loss and 76% of 150 patients who underwent surgery for weight loss [32]. A 17% reduction in body weight was considered to be required to achieve a clinically meaningful improvement [32]. In the current study, a participant in the LEPR trial with a body weight change of − 15.6% did not meet this − 17% threshold but had a greater improvement in IWQOL-Lite total score (change of 21; clinically meaningful improvement) than expected. While just 1 patient, this might suggest that weight loss is not the only factor driving improvement in IWQOL-Lite total scores; notably, this patient experienced a 67.9% reduction in “most” hunger score.

Children and adolescents with obesity due to POMC or LEPR deficiency in these studies tended to have more impaired baseline QOL (mean [SD] PedsQOL total scores 53.3 [6.2] for children and 63.3 [29.1] for adolescents) than children with other chronic conditions, such as diabetes mellitus, cardiac disease and cancer, from other studies, with mean (SD) PedsQL total scores 80.4 (12.9), 77.5 (14.5), and 72.0 (16.1), respectively [33]. Clinically meaningful improvements in PedsQL total, physical functioning, and psychosocial health scores were observed for some children and adolescents.

Effects of setmelanotide on patient QOL were seen relatively quickly (as soon as Week 5) and were maintained throughout the study. Continued improvement in QOL may be diminished or delayed by certain family situations. Some patients have experienced a difficult relationship with their parents that had lasting effects because of their obesity [1]. Some patients may even worry about the burden they place on their family or caregivers [18]. Family stressors may lead to further weight gain in pediatric patients [16].

While depression as measured by the PHQ-9 is not a direct QOL measure, PHQ-9 scores have been shown to negatively correlate with QOL [34]. Some individuals with POMC or LEPR deficiency experienced mild-to-moderate depression at the time of trial enrollment. This may stem from the burden of their disease. While moderately severe or severe depression (PHQ-9 score ≥ 15) was an exclusion criterion, no patients were excluded or withdrew from the study based on PHQ-9 scores, suggesting that the reported PHQ-9 scores were representative of the patient population. Centrally acting antiobesity therapeutics may be associated with neuropsychiatric adverse effects, including depression, as was seen with rimonabant treatment and others [35]. Levels of patient depression were generally consistent with baseline at Week 52, suggesting no worsening of depressive symptoms with setmelanotide treatment.

These two pivotal Phase 3 trials of setmelanotide were limited by the low prevalence of the extremely rare diseases evaluated, the separation of the enrolled patients by age group according to the questionnaires available, and incomplete responses on some questionnaires. Furthermore, the data are based in part on the IWQOL-Lite, which is not tailored to patients in obesity clinical trials [36]. The IWQOL-Lite-CT may be a more sensitive measure for this patient population [36]. Clinically meaningful changes in patient-reported PedsQL scores from a control population of children were used as the reference for both the child and adolescent populations in this study, given that adolescent scores were not available. The comparator population used to define clinically meaningful changes could affect the interpretation of QOL results. However, in this study, patients who experienced a clinically meaningful change often had scores well beyond the minimally defined thresholds referenced. Finally, this trial assessed disease-specific QOL measures for 1 year, which may be an insufficient amount of time for patients to overcome burdens that they may have lived with for multiple years.

However, these data, although originating from patients with differing ages, demonstrate the burden of the diseases, which are also characterized by hyperphagia and early-onset severe obesity. The previous results of the Phase 3 trial and additional Phase 2 trials provide evidence that treatment with setmelanotide reduces hunger scores [20, 21, 37]. Some patients saw only moderate effects on QOL, which may indicate that in some patients there are long-lasting individual consequences that alter the effect of hyperphagia including intrafamilial dynamics, education, and social environment. However, despite QOL being a complex metric impacted by many variables, some patients improved to no impairment in QOL after setmelanotide treatment. Getting control over one part of the disease is not sufficient, at least over ~ 52 weeks, to normalize QOL. The results of this study indicate that it is essential to support patients and their families psychologically, independent of pharmacologic treatment. Further, psychological support is warranted during pharmacologic treatment, such as treatment with setmelanotide, which can be the driver for weight loss in some patients. It might take longer than 52 weeks to normalize QOL, given that some patients have lived with their disease since birth [22]. It will be of interest to evaluate whether setmelanotide treatment from an early age (e.g., before the age of 3 years) might be likely to avoid the occurrence of any psychosocial symptoms.

## Conclusions

This study demonstrated that patients with POMC or LEPR deficiency have impaired QOL, which may improve with setmelanotide treatment. Setmelanotide, through its reduction in hunger and associated weight loss, may have a meaningful impact on patient QOL, especially when combined with interdisciplinary support in experienced centers. The psychological consequences of rare genetic diseases of obesity are highly complex, and support by trained psychologists should be implemented in the treatment strategy.

## Data Availability

The data sets used and/or analyzed during the current study are available from the corresponding author on reasonable request.

## Abbreviations

BMI: Body mass index
DUS: Designated use set
FAS: Full analysis set
IWQOL-Lite: Impact of Weight on Quality of Life-Lite
POMC: Proopiomelanocortin
LEPR: Leptin receptor
QOL: Quality of life
PedsQL: Pediatric Quality of Life Inventory
PHQ-9: Patient Health Questionnaire-9
SD: Standard deviation
